# Predictors of Pregnancy-Related Emotions

**DOI:** 10.4021/jocmr1246e

**Published:** 2013-02-25

**Authors:** Mojmir Tyrlik, Stepan Konecny, Lubomir Kukla

**Affiliations:** a Institute for Research on Children, Youth and Family. Masaryk University - Faculty of Social Studies. Brno. Czech Republic; b Faculty of Business and Management - Brno University of Technology. Brno. Czech Republic; c Research Institution of Preventive and Social Paediatrics. Masaryk University - Faculty of Medicine. Brno. Czech Republic

**Keywords:** Emotions in pregnancy, Health status, Partnership, Social support, Planned pregnancy

## Abstract

**Background:**

The study explores the pregnancy-related emotions of women. The investigated predictive factors include the woman’s age, previous maternal experiences, especially miscarriage or birth defects, skills related to maternity, pregnancy planning, objective and subjective health status, social relationships and social support, especially the partner relationship, and housing status.

**Methods:**

The Czech ELSPAC data obtained from 4,890 pregnant women was used.

**Results:**

Age, partnership, previous pregnancy experience, pregnancy planning, and standard of housing all relate significantly to the emotions in the first month and in the sixth month of pregnancy. A change in the mother’s emotional experience during pregnancy is significantly predicted by subjective health and social support.

**Conclusions:**

Health, social relationships, material conditions, and psychological preparedness affect the positive emotional experience of pregnancy. Women who planned to become pregnant are more content. However, the overall emotional experience also relates to the social and psychological preparedness for the upcoming changes.

## Introduction

Pregnancy and childbirth are unquestionably important moments in the life of a woman. Many women around the world look forward to having children and experience pregnancy positively in general. On the other hand, there are some psychological dispositions and life conditions which occasionally cause negative emotions in the mother. The study explores and attempts to explain the pregnancy-related emotions of women. These emotions substantially influence behaviour during pregnancy and after delivery.

The factors related to the development of pregnancy and maternity that was investigated were primarily emotions and moods, especially their pathological poles, such as anxiety, depression, and neuroticism, objective and subjective health status, social relationships, particularly the partner relationship, and economic status [1-4]. The woman’s previous maternal experience, especially miscarriage or birth defects, and skills related to pregnancy, labour, and maternity are also important factors. Whether the pregnancy was planned or desired also constitutes a specific motivational factor [5-7].

We can combine the personal and social issues into at least three factors underlying the emotions. The first factor concerns the personality conditions related to the woman’s ability to cope with burden, especially her emotional stability and anxiety, as well as the subjective and objective health status of the woman and of the foetus during development. Depression and anxiety are the most frequently evaluated psychological risk factors in the literature. These factors can originate from (and manifest in) inadequate management of various pregnancy-related difficulties, such as nausea, fear for the foetus, employment and social limitations, and so on. The mood state can moderate or amplify the effect of other factors which in turn influence the emotional state. For example, anxiety is associated with the anticipation of less control and support while giving birth [5]. On the other hand, a positive, optimistic personality can soften or repress inconveniences, and a mother’s secure attachment can influence the experience of pregnancy positively at the very beginning [8-11]. Health problems, especially those that occur during pregnancy, can result in physical complaints and worries about the foetus [12]. Despite some health problems being objective and considerable, every woman varies in the experience of these problems due to her specific perceptions, which could be individually amplified, moderated, or ignored, based on a woman’s personality disposition and experience in general [13]. In other words, the mother’s consideration and feelings transfer physically objective symptoms as well as any suspicious somatic phenomena into the subject of ‘subjective health’.

The second factor includes the maternal plans and skills of the woman. A very important aspect is the woman’s previous maternal experience, especially miscarriage or birth defects. The results of previous pregnancies provide a vivid experience imprinted in the mother’s memory. The multiparous are capable of handling many relevant situations better than the nulliparous. Matching expectations to reality is expected to improve the experience and reduce pain during delivery [5, 14]. On the other hand, there are many women who have had an adverse experience, giving birth to a dead child or a child who died afterwards. Abortion can be included among adverse experiences as well. These women could view their present pregnancy from the perspective of previous traumatic experience [15].

The third factor relates to social and material life conditions. It includes close relationships and the necessary social support promising a satisfactory course of pregnancy and future maternity as well as material or economic status, continuous employment, and acceptable housing standards. With respect to social factors, a partnership should first be taken into account. A partner’s support is important for effective coping with stressful events [16]. Conflict with her partner, fear of her partner leaving her, or her partner’s aggressive behaviour towards her exhausts a pregnant woman psychologically as well as physically and increases her insecurity regarding the situation after birth. Social support from family, relatives, friends, or work colleagues may also be considered, depending on cultural circumstances [1, 4].

Some researchers assume that each of these factors causes separate feelings [17]. For instance, a mother could be satisfied with her housing or financial standard while simultaneously being afraid about the future development of the relationship with her partner. However, we would theorize about a single general pregnancy-related emotion, which can overwhelm or moderate any other emotions. It does not matter whether this emotion originates from previous traumatic experience, employment, or another factor.

Our research aims to explore the effect of the above-mentioned factors on the general experience of pregnancy at the very beginning and its changes up to the sixth month. We also followed the factors as predictors of experience development during the first six months of pregnancy.

## Material and Methods

Data was obtained from the European Longitudinal Study of Pregnancy and Childhood (ELSPAC), a prospective study observing a set of children and their families from the pregnancy through delivery, maternity leave, and childhood until the children reached at least 18 years of age. The Czech sample includes children (and their parents) who were born in the city of Brno (approx. 4,500) and the rural district of Znojmo (approx. 1,500) between March 1, 1991, and June 30, 1992. We used the available data of 5,019 pregnant women in their sixth month of pregnancy.

Extensive questionnaires were sent by mail to pregnant women. The women were asked to recall the emotions they experienced when they first received medical confirmation of pregnancy. They then evaluated their present pregnancy-related emotions (in the sixth month of pregnancy). A five-point ordinary scale captured ‘very happy’, ‘glad’, ‘ambiguous emotions’, ‘unhappy’, and ‘very unhappy’ with an additional point for ‘without emotions’. For further analysis, we excluded the sixth additional point, which did not refer to the emotional modality. We divided the values of the five-point ordinary scale into two categories. One category consisted of positive emotions (former values one and two) (3,478 (68.7%) of the women at the first month and 4,226 (85.3%) of the women at the sixth month, respectively); the second category consisted of negative or ambiguous emotions (1,581 (31.3%) and 728 (14.7%), respectively).

The sample was divided into three age groups: under 21 (725 (17.9%)), ages 21 to 30 (2,696 (66.5%)), and over 30 (634 (15.6%)). With respect to previous pregnancy experiences and outcomes, we divided the sample into women who already have a child (2,410 (48.3%)) and those who do not. The second group was further divided into two subgroups. The first subgroup was women who had had a miscarriage or stillbirth, or had had a child who died immediately after delivery (623 (12.5%)). Women who had undergone an abortion in the past were included in this group. The second subgroup consisted of women without previous trauma related to pregnancy (1959 (39.2%)).

The Crown-Crisp Experiential Index (CCEI) was used to evaluate neurotic symptoms [18]. We used it as a scale variable as well as a nominal variable in a later analysis. In the later analysis, the values were divided with respect to magnitude of neurosis to the 85th percentile.

The women were classified into three groups with respect to their relationships with partners. The first group was women with a ‘healthy’ partnership, the second was women who reported a problem with their partner, and the third was women without a partner.

We used a social support scale which consists of a 10-item set of questions that identify the extent of a woman’s social support - a network that enables communication and the resolution of life problems. We divided the women into two groups according to a 5.4 percentile in order to amplify the lack of social support. Women who reached a cumulative value of 8 to 10 were selected as those with satisfactory social support; others were classified as having ‘low social support’.

We considered whether medical treatment was necessary for any of 15 specific health problems during pregnancy for examining the ‘objective health’ of each woman. A woman was considered ‘objectively healthy’ if there was no treatment. A woman was classified ‘objectively not healthy’ if treatment for at least one condition occurred.

A five-point ordinary scale was used to assess the subjective health of women at the beginning and in the sixth month of pregnancy. The poles of the scale were labelled ‘absolutely excellent’ and ‘absolutely bad’. We divided the sample according to the scale value into two groups. The two higher points were recorded as a new value ‘no health complaints’; the rest were classified as ‘health complaints’.

We also split the women according to the standard of housing. Temporary or provisional housing was considered to be low standard.

We compared the groups that were divided according to the variables stated above with respect to the categories of emotions which women experienced both at the beginning of pregnancy and in the sixth month of pregnancy. The variables were used as consecutive covariates in a logistic regression model in order to predict emotions in each period. Finally, we used the covariates to predict emotional changes during pregnancy. We used the SPSS 16 chi-square test and binary logistic regression. The frequencies of subjects in individual computations depend on the frequencies of cumulative missing values in the used scales.

## Results

Age, partnership, previous pregnancy experience, planned pregnancy, and standard of housing are significantly related to the emotions at the first moment of pregnancy confirmation (first month), as recalled by mothers during the sixth month of pregnancy. The emotions in the sixth month were related to all of these factors (except for the standard of housing), extended by social support, subjective evaluation of health, and the presence of medically treated problems (objective health) (Table 1).

**Table 1:** Percentage of Women With Positive Emotions

Variable	N	Category	Percentage of positive emotions		1st month	6th month	
			1st month	6th month		
age	600	over 30 yrs	64.3	78.3	χ^2^ (d.f.= 2) = 24.2***	χ^2^ (d.f.= 2) = 31.8***
	2,572	21 yrs - 31 yrs	72.1	87		
	698	under 21 yrs	64.8	86.1		
partnership	425	problems with partner	54.4	79.8	χ^2^ (d.f.= 2) = 89.6***	χ^2^ (d.f. = 2) = 45.2***
	70	has no partner	41.4	61.4		
	4,345	“healthy” partnership	71.5	86.3		
previous pregnancy experiences	598	trauma	71.2	85.6	χ^2^ (d.f. = 2) = 14.2***	χ^2^ (d.f. = 2) = 24.8***
	1,890	primigravida	71.9	88.2		
	2,274	has a child	67.1	82.8		
social support	230	low	68.7	77.8	n.s.	χ^2^ (d.f. = 1) = 9.7**
	4,211	High	69.7	85.8		
objective health	2,244	no treatment	70.8	87.3	n.s.	χ^2^ (d.f. = 1) = 6.4*
	2,043	treatment	69	84.6		
subjective health	3,330	no complaints	70.1	87.5	n.s.	χ^2^ (d.f. = 1) = 47.4***
	1,181	complaints about health	66.9	78.4		
planned pregnancy	2,421	planned	91.4	89.5	χ^2^ (d.f. = 1) = 1150.9***	χ^2^ (d.f. = 1) = 67.3***
	2,414	Unplanned	47.5	81.2		
housing	2,019	sub-standard	66.5	84.6	χ^2^ (d.f. = 1) = 14.9***	n.s.
	2,753	standard	71.7	86		

*** significant at P < 0.001; ** significant at P < 0.01; * significant at P < 0.05.

The emotions changed significantly over the course of pregnancy. There was emotional improvement in 1,015 initially unhappy woman (20.8%), and worsening in 240 initially happy women (4.9%); 475 unhappy women (9.7%) and 3,160 happy women (64.6%) (χ^2^ (d.f. = 1) = 511.24; P < 0.001) were unchanged.

All of the variables were significantly related to the change of emotions between the first month and the sixth month of pregnancy. A considerable drop in positive emotions was observed in the group of mothers without partners (11.4%) and the group of women who did not feel healthy (7.4%).

The highest percentages of improved pregnancy-related emotions were in the group who did not plan their pregnancy (36.8%) and the group without partners (31.4%). The least improvement was seen in the group of women without social support (Table 2).

**Table 2:** Percentage of Women Who Changed Emotionally From 1st to 6th Month

Variable	N	Category	Emotions		
			Deterioration (%)	Improvement (%)
age	600	over 30 yrs	6.3	20.3	χ^2^ (d.f. = 2) = 24.2***
	2,572	21 yrs - 31 yrs	4.3	19.2	
	698	under 21 yrs	5	26.4	
partnership	425	problems with partner	4.7	30.1	χ^2^ (d.f. = 7) = 524.4***
	70	has no partner	11.4	31.4	
	4,345	“healthy” partnership	4.8	19.6	
previous pregnancy experience	598	trauma	4.2	18.6	χ^2^ (d.f. = 7) = 385.5***
	1,890	primigravida	4.9	21.2	
	2,274	has a child	5.2	20.9	
social support	230	low	6.5	15.7	χ^2^ (d.f. = 4) = 336.5***
	2,933	high	4.9	21.1	
objective health	2,244	no treatment	3.8	20.4	χ^2^ (d.f .= 4) = 417.4***
	2,043	treatment	5.6	21.2	
subjective health	3,330	no complaints	4	21.5	χ^2^ (d.f. = 4) = 567.1***
	1,181	complaints about health	7.4	18.9	
planned pregnancy	2,421	planned	6.7	4.9	χ^2^ (d.f. = 4) = 1,705.8***
	2,414	unplanned	3.1	36.8	
housing	2,019	sub-standard	5	23.1	χ^2^ (d.f. = 4) = 472.4***
	2,753	standard	4.8	19.1	

*** significant at P < 0.001; ** significant at P < 0.01; * significant at P < 0.05.

Logistic regression was used to evaluate the predictors of emotions in both the first and the sixth month of pregnancy.

The variables were entered in three blocks. The first block (A) consisted of the neurotic symptoms measured by the CCEI, subjective evaluation of health, and presence of medically treated problems (objective health). The second (B) consisted of age, outcome of previous pregnancies, and planning of current pregnancy. The third (C) was included quality of partnership, social support, and housing standard. We chose certain functionally normal categories in each of the variables as the referential points of comparison. These selected referential categories were: ages 21 to 30, already have a child, planned the current pregnancy, with a regular partner, have no disease and consider themselves healthy, with satisfactory social support, and with standard housing conditions.

Every one of the three blocks of variables (Table 3) significantly predicted the emotional experiences at the first medical confirmation of pregnancy. The CCEI is a significant predictor in block A. The planning of pregnancy and previous pregnancy outcomes are the most powerful predictors in block B. The odds of positive emotions in the group of women who did not deliberately become pregnant are 0.068 times the odds in the group of women who got pregnant deliberately. This result is extremely significant. The odds of positive emotions are also significantly higher in the group of primiparous women than the group who already have a child. The odds are lower in the age group over 30 years at a less significant level.

**Table 3:** Binary Logistics Regressions Results - 1st Month

Block		B	S.E.	Wald	df	Sig.	Exp (B)
a	CCEI	-0.034	0.006	33.948	1	0.000	0.967
a	objective health (treatment)	-0.132	0.098	1.827	1	0.176	0.876
a	subjective health (complaints)	0.016	0.100	0.025	1	0.876	1.016
b	age			7.218	2	0.027	
	age (over 30 yrs)	-0.295	0.139	4.529	1	0.033	0.744
	age (under 21 yrs)	0.191	0.136	1.984	1	0.159	1.211
b	previous pregnancy experience (PPE)			27.789	2	0.000	
	PPE (trauma)	0.175	0.157	1.242	1	0.265	1.191
	PPE (primigravida)	0.607	0.116	27.233	1	0.000	1.836
b	planned pregnancy (unplanned)	-2.690	0.115	548.877	1	0.000	0.068
c	partnership			13.515	2	0.001	
	partnership (problems w. partner)	-0.507	0.172	8.674	1	0.003	0.602
	partnership (has no partner)	-0.908	0.377	5.802	1	0.016	0.403
c	social support (low)	0.194	0.224	0.745	1	0.388	1.214
c	housing (sub standard)	-0.036	0.106	0.112	1	0.738	0.965
	Constant	3.688	0.246	224.534	1	0.000	39.956

Block a: the CCEI scale, objective health, subjective health: Hosmer Lemeshow test (χ^2^ d.f. = 8) = 6.84; P = 0.554; Omnibus test (χ^2^ d.f. = 4) = 44.83; P < 0.001; Nagelkerke R-Square = 0.021; Block b: age category, previous pregnancy status, planned pregnancy: Hosmer Lemeshow test (χ^2^ d.f. = 8) = 4.10; P = 0.848; Omnibus test (χ^2^ d.f. = 5) = 823.35; P < 0.001; Nagelkerke R-Square = 0.362; Block c: partnership, social support, standard of housing: Hosmer Lemeshow test (χ^2^ d.f. = 8) = 4.36; P = 0.822; Omnibus test (χ^2^ d.f. = 4) = 15.01; P = 0.005; Nagelkerke R-Square = 0.367.

In block C, problems with partners significantly lower the odds of positive emotions.

The three blocks of variables also significantly predicted emotions during the sixth month of pregnancy (Table 4). In block A, in addition to the already significant CCEI, subjective health also became a significant predictor. The odds of positive emotions in the group of women who complained about health are 0.718 times the odds of women who did not complain. In the age group over 30, the ratio is significantly lower than with women between 21 and 30 years of age. Simultaneously, as in the first month, we can see higher odds of positive emotions in the group of primiparous women and women with previous traumatic pregnancy experience than in the group of women who already have a child.

**Table 4:** Binary Logistics Regressions Results - 6th Month

Block		B	S.E.	Wald	df	Sig.	Exp (B)
a	CCEI	-0.053	0.007	62.296	1	0.000	0.948
a	objective health (treatment)	-0.106	0.122	0.755	1	0.385	0.900
a	subjective health (complaints)	-0.332	0.134	6.160	1	0.013	0.718
b	Age			24.769	2	0.000	
	age (over 30 yrs)	-0.682	0.150	20.591	1	0.000	0.506
	age (under 21 yrs)	0.286	0.191	2.227	1	0.136	1.330
b	previous pregnancy experience (PPE)			17.039	2	0.000	
	PPE (trauma)	0.391	0.199	3.869	1	0.049	1.478
	PPE (primigravida)	0.619	0.152	16.585	1	0.000	1.857
b	planned pregnancy (unplanned)	-0.784	0.125	39.310	1	0.000	0.456
c	partnership			21.291	2	0.000	
	partnership (problems w. partner)	-0.417	0.211	3.913	1	0.048	0.659
	partnership (has no partner)	-1.624	0.372	19.107	1	0.000	0.197
c	social support (low)	-0.242	0.247	0.955	1	0.328	0.785
c	housing (sub standard)	-0.337	0.133	6.407	1	0.011	0.714
	Constant	4.612	0.293	248.450	1	0.000	100.709

Block a: the CCEI scale, objective health, subjective health: Hosmer Lemeshow test (χ^2^ d.f. = 8) = 6.14; P = 0.632; Omnibus test (χ^2^ d.f. = 4) = 106.185; P < 0.001; Nagelkerke R-Square = 0.07; Block b: age category, previous pregnancy status, planned pregnancy: Hosmer Lemeshow test (χ^2^ d.f. = 8) = 8.05; P = 0.428; Omnibus test (χ^2^ d.f. = 5) = 90.306; P < 0.001; Nagelkerke R-Square = 0.127; Block c: partnership, social support, standard of housing: Hosmer Lemeshow test (χ^2^ d.f. = 8) = 3.61; P = 0.890; Omnibus test (χ^2^ d.f. = 4) = 29.740; P < 0.001; Nagelkerke R-Square = 0.146.

Although the planning of pregnancy remains a significant predictor, the Wald statistic is not as extreme as in the first month. Problematic partnerships and a lower standard of housing significantly lower the probability of positive emotions.

We created a new binary ‘change variable’ in order to evaluate emotional change from the first to the sixth month of pregnancy. A low value represents worsening of emotions; a higher value represents improvement. Worsening or improvement of the emotional experience during pregnancy is significantly predicted only by the first two blocks of variables; only two factors significantly affected mood changes: subjective health and accessibility of social support (Table 5). While the occurrence of objective health problems is rather neutral, subjective health concerns are a significant predictor of negative emotions. This phenomenon cannot be fully linked to neurotic symptoms since the average CCEI range is comparable to the group with no neurotic indications and we observed only a small, insignificant shift towards worse experience in the group with peripheral CCEI values. We have also found a significantly higher probability of improvement in previously frequently unhappy groups: the group of women who did not plan their conception, as well as an insignificantly higher probability of improvement in the group of traumatized women, women with problematic partnerships, and women without a partner.

**Table 5:** Binary Logistics Regressions Results - the Change in Emotions From 1st to 6th Month

Block		B	S.E.	Wald	df	Sig.	Exp (B)
a	objective health (treatment)	-0.101	0.256	0.156	1	0.693	0.904
a	subjective health (complaints)	-0.729	0.276	6.988	1	0.008	0.482
a	CCEI			1.436	2	0.488	
	CCEI middle range	0.011	0.363	0.001	1	0.977	1.011
	CCEI neurotics	-0.357	0.429	0.690	1	0.406	0.700
b	age			2.277	2	0.320	
	age (over 30 yrs)	-0.500	0.335	2.222	1	0.136	0.607
	age (under 21 yrs)	-0.144	0.366	0.155	1	0.694	0.866
b	previous pregnancy experience (PPE)			2.730	2	0.255	
	PPE (trauma)	0.582	0.469	1.541	1	0.214	1.790
	PPE (primigravida)	-0.193	0.299	0.416	1	0.519	0.825
b	planned pregnancy (unplanned)	2.688	0.257	109.543	1	0.000	14.702
c	partnership			3.466	2	0.177	
	partnership (problems w. partner)	0.350	0.470	0.555	1	0.456	1.420
	partnership (has no partner)	-1.116	0.686	2.651	1	0.104	0.328
c	housing (sub standard)	-0.226	0.268	0.710	1	0.400	0.798
c	social support (low)	-1.275	0.524	5.910	1	0.015	0.280
	Constant	0.471	0.392	1.442	1	0.230	1.601

Block a: the CCEI scale, objective health, subjective health: Hosmer Lemeshow test χ^2^ (d.f. = 8) = 0,852; P = 0.991; Omnibus test (χ^2^ d.f. = 4) = 13,701; P < 0.01; Nagelkerke R-Square = 0.035; Block b: age category, previous pregnancy status, planned pregnancy: Hosmer Lemeshow test (χ^2^ d.f. = 8) = 13,124.05; P = 0.108; Omnibus test (χ^2^ d.f. = 5) = 127,137; P < 0.001; Nagelkerke R-Square = 0.328; Block c: partnership, social support, standard of housing: Hosmer Lemeshow test (χ^2^ d.f. = 8) = 5,884; P = 0.660; Omnibus test (χ^2^ d.f. = 4) = 9,419; P = 0.051; Nagelkerke R-Square = 0.348.

## Discussion

The results confirm the assumption that the age and health of a woman, her psychological preparedness for maternity, partnership status and social relationships, and some material conditions significantly affect the emotional experience of pregnancy.

At the beginning of pregnancy, a satisfactory relationship with a partner and overall preparedness for pregnancy, as indicated by a planned conception, evoke positive emotions. Mothers also tend to be most content with their pregnancy when they wanted to become pregnant. This is not only related to a positive acknowledgement of pregnancy as the impending arrival of a wanted child, but also with the overall social and psychological preparedness for the upcoming changes. Barrett and Wellings refer to a broadened understanding of planned pregnancy in British women [19]. Our data support the generally accepted role of satisfactory partnership and social support in this context [4].

The importance of planned conception gradually decreases during pregnancy, although it remains a significant predictor of positive emotional experiences over the first six months of pregnancy.

The second half of pregnancy is affected more by the mother’s health, especially her subjective assessment of her own health, and by the social support provided by family and friends. Although the experience of the beginning of pregnancy is mostly affected by the objective adverse living and personal conditions of the woman, many women find a way to cope with these conditions in the second half of pregnancy, either alone or with the help of friends. This is also indicated by the ratio of women whose experience of pregnancy improved dramatically during the course of pregnancy. Although significant changes in the number of happy and unhappy women occurred in all of the studied groups, the largest increase, of 36%, occurred in the group of women who did not plan the conception. A significant improvement was also found in the group of women under 21 years of age and in the group of women without a partner or with problems in their relationship. On the other hand, more than 10% of women who became unhappy during pregnancy were in the group of women without a partner. Furthermore, the number of initially happy women also strongly decreased in the group of women with low social support, and women who subjectively believed they had health problems. The experience of pregnancy also became negative in the sixth month for 6.7% in the group of women who planned their conception as well. These findings are in concordance with results of Glazier et al, who found that social plays a mediating role on emotional distress in pregnant women [20].

The high emotional improvement in the group with an unplanned pregnancy is probably related to a significant increase in the number of content women among those who were originally surprised by the pregnancy but later viewed it as a joyful event. This finding fits the assumption that emotional response changes as pregnancy advances and life events that are experienced later in pregnancy are perceived as less stressful than those that occur at the beginning [21]. On the other hand, the considerable percentage of women who became less happy or unhappy during the pregnancy shows the vulnerability of those who underestimate pregnancy-related stress.

The neurotic symptoms measured by the CCEI are a significant predictor of experiencing pregnancy in the first and sixth month of pregnancy, and its influence in the second period increases slightly. Its influence is quite limited when compared to other factors. Nevertheless, our findings support the assumption of a connection between neuroses and stressful or dangerous factors in pregnancy [5]. It is noteworthy that women with a traumatic experience from previous childbirth do not experience their first month differently than those who are pregnant for the first time. However, the increase of happy women in their sixth month of pregnancy is lower in the group with a traumatic experience than in the group experiencing pregnancy for the first time.

Our results indicate that the way women experience pregnancy changes during the course of pregnancy in approximately 25% of women. Around 20% of women experience an increase in happiness while 5% experience a decrease in happiness. Positive changes in objectively at-risk groups (for example, with health, housing, or relationship problems) indicate that the improvement is likely attributed to the increased or asserted capability of these women to cope with objective problems.

The data we have used were obtained in the 1990s. There are unquestionably some aspects of pregnancy which have changed since then. For instance, the average age of primiparas has increased. On the other hand, the factors studied mostly relate to basic human needs (for example, social support, housing, health, and family planning). We therefore hypothesize timeless relationship between these factors and the emotional experience of pregnancy, excluding an extreme and abnormal situation when satisfaction of a need is dramatically challenged.
